# Iteration method for predicting essential proteins based on orthology and protein-protein interaction networks

**DOI:** 10.1186/1752-0509-6-87

**Published:** 2012-07-18

**Authors:** Wei Peng, Jianxin Wang, Weiping Wang, Qing Liu, Fang-Xiang Wu, Yi Pan

**Affiliations:** 1 School of Information Science and Engineering, Central South University, Changsha, Hunan 410083, People’s Republic of China; 2 Computer Technology Application Key Lab of Yunnan Province, Kunming University of Science and Technology, Kunming, Yunnan, 650093, People’s Republic of China; 3 Department of Mechanical Engineering and Division of Biomedical Engineering, University of Saskatchewan, Saskatoon, SK S7N 5A9, Canada; 4 Department of Computer Science, Georgia State University, Atlanta, GA, 30302-4110, USA

## Abstract

**Background:**

Identification of essential proteins plays a significant role in understanding minimal requirements for the cellular survival and development. Many computational methods have been proposed for predicting essential proteins by using the topological features of protein-protein interaction (PPI) networks. However, most of these methods ignored intrinsic biological meaning of proteins. Moreover, PPI data contains many false positives and false negatives. To overcome these limitations, recently many research groups have started to focus on identification of essential proteins by integrating PPI networks with other biological information. However, none of their methods has widely been acknowledged.

**Results:**

By considering the facts that essential proteins are more evolutionarily conserved than nonessential proteins and essential proteins frequently bind each other, we propose an iteration method for predicting essential proteins by integrating the orthology with PPI networks, named by ION. Differently from other methods, ION identifies essential proteins depending on not only the connections between proteins but also their orthologous properties and features of their neighbors. ION is implemented to predict essential proteins in S. cerevisiae. Experimental results show that ION can achieve higher identification accuracy than eight other existing centrality methods in terms of area under the curve (AUC). Moreover, ION identifies a large amount of essential proteins which have been ignored by eight other existing centrality methods because of their low-connectivity. Many proteins ranked in top 100 by ION are both essential and belong to the complexes with certain biological functions. Furthermore, no matter how many reference organisms were selected, ION outperforms all eight other existing centrality methods. While using as many as possible reference organisms can improve the performance of ION. Additionally, ION also shows good prediction performance in *E. coli* K-12.

**Conclusions:**

The accuracy of predicting essential proteins can be improved by integrating the orthology with PPI networks.

## Background

Essential proteins (also known as lethal proteins) are indispensable to life as without them the lethality or infertility is caused. Identification of essential proteins has been the pursuit of biologists for two main purposes. From the theoretical perspective, identification of essential proteins provides insight in understanding minimal requirements for cellular survival and development. It also plays a significant role in the emerging science of synthetic biology which aims to create a cell with minimal genome [1]. From the practical perspective, essential proteins are drug targets for new antibiotics, due to their indispensability for bacterial cell survival [2]. Moreover, research results suggest that essential proteins (or genes) have associations with human disease genes [3]. Studying of essential proteins also facilitates identifying the disease genes. In biology, there are many experimental methods which can predict and discover essential proteins, such as single gene knockouts [4], RNA interference [5] and conditional knockouts [6]. However, these experiments are expensive and inefficient. Furthermore, they are limited to a few species. So a highly accurate computational method becomes a very important choice for identifying essential proteins.

Recently, many computational methods have been proposed to identify essential proteins, based on the features of essential proteins. One of the most important features of essential proteins is their conservative property. Previous studies have shown that essential proteins evolve much slower than other proteins. They are more evolutionarily conserved than nonessential proteins [7-9]. This is because essential genes are more likely involved in basic cellular processes, thus the negative selection acting on essential genes are more stringent than non-essentials [8]. In several studies [10-12], the term ‘phyletic retention’ is introduced to describe the homology mapping of a protein in other organisms by using BLAST or reciprocal best hit [12], in place of the term ‘conservation’. Moreover, Gustafson et al. [10] point out the phyletic retention is the most predictive of essentiality. Besides the phyletic retention trait of proteins, other types of genomic features, such as GC content, protein length, ORF length [10-12], cellular localization [13], and so on, are also mentioned for predicting essential proteins by taking the advantage of supervised machine learning-based methods. Since these methods develop a classifier to learn traits of essential genes in one organism and then predict those in the other organism or in the test dataset of the same organism, a set of essential proteins and their related properties have to be known in prior. Consequently, the performance of these methods closely depends on classifier and the distance between training organisms and test organisms.

Another important feature of essential proteins is their topological properties in Protein-Protein Interaction (PPI) networks. Proteins in cells interact with each other and construct a PPI network. A group of researchers focus on studying the relationships between essentialities and topological properties of proteins in PPI networks. Study has shown that there is a positive correlation between the lethality and the centrality in PPI networks [14]. Thus, the most highly connected proteins are more likely to be indispensable. As a consequence, a series of centrality measures based on network topological features have been used for identifying essential proteins, such as Degree Centrality (DC) [15], Betweenness Centrality (BC) [16] Closeness Centrality (CC) [17], Subgraph Centrality (SC) [18], Eigenvector Centrality (EC) [19], Information Centrality (IC) [20] and Edge Clustering Coefficient Centrality (NC) [21] and so on. These methods rank proteins in terms of their centrality in PPI networks. Then the ranking scores of these proteins are used to judge whether a protein is essential. The merit of these methods is that they identify essential proteins directly and don’t need to train a classifier according to a set of known essential proteins.

However, there exist some limitations on these centrality methods. Firstly, the available PPI data is incomplete and contains many false positives and false negatives, which impacts the correctness of discovering essential proteins. Secondly, most of these methods seldom analyze other intrinsic properties of the known essential proteins while using only topological properties of networks. To overcome these limitations, recently many research groups have focused on identification of essential proteins by integrating PPI networks with other biological information. Li et al. [22] construct a weighted PPI network by taking consideration of gene annotations. With the integration of network topology and gene expression, the same group of researchers proposes a new method called PeC [23]which increases the predictability of essential proteins in comparison with those centrality measures only based on network topological features. On the other hand, by using supervised machine learning-based methods, some researchers combine network topological properties with genomic features, such as cellular localization [13] to identify essential proteins.

Additionally, Pereira-Leal et al. [24] have reported that essential proteins are, on average, more frequently connected to other essential proteins than nonessential proteins are. By analyzing the topological properties of interactions between essential proteins, they have detected an almost fully connected exponential network, which implies a strong correlation between the essentiality of a protein and that of its neighbors.

Based on the facts mentioned above, we propose an iteration method for predicting essential proteins by integrating orthology with PPI network, named as ION. In ION, the conservative property of proteins is also taken into account. To measure the conservation of proteins, we find orthologous proteins in other species, instead of sequence alignment using BLAST. Orthologs are homologous proteins that are derived from a common ancestor. They usually have high similar amino acid sequences and retain the same or very similar functions. This allows us to infer biological information between these proteins. Many studies use orthologous information to identify evolutionary signals of PPI networks [25,26], discover the rate of protein evolution [27], infer protein conservation [28,29]. Recently more and more algorithms have been used to detect orthologs, such as IsoRank [30,31]. Furthermore, many databases and public resources of orthologs are available now, for instance, COG [32], ORTHOMCL [33], OMA [34], IsoBase [35] and Inparanoid [36], which facilitate orthologs-based researches.

In addition to orthologous properties of proteins, the connectivity and features of their neighbors are also considered in ION. Comparing with supervised machine learning methods, ION combines the three features to give each protein a ranking score using the iteration method without knowing a set of essential proteins. Differently from other centrality methods, ION identifies essential proteins depending on not only the connections between proteins but also their orthologous properties and features of their neighbors. To evaluate the performance of ION, we predict essential proteins by using yeast data sets. Experimental results show that the prediction performance of ION by integrating the proteins’ orthologous property with their neighbor’s property in the PPI network is better than that by using only either property. Moreover ION can achieve better performance in essentiality prediction than above eight other existing centrality methods (DC, BC, CC, SC, EC, IC, NC and PeC) in terms of their precision-recall (PR) curves and jackknife curves. In top 100 ranked proteins, ION identifies 78 essential proteins and NC only identifies 55 essential proteins, which illustrates that ION achieves 42% improvement than NC that has the best performance among the seven existing centrality methods(DC, BC, CC, SC, EC, IC and NC). Compared with PeC which identifies essential proteins by integrating gene expression data with PPI networks, ION also outperforms it. Especially, with more candidate proteins selected, the advantage of ION in the prediction of essential proteins becomes increasingly obvious. Moreover, compared with PeC, NC and DC, more proteins in top 100 ranked by ION belong to the complexes with certain biological functions. In order to investigate whether the amount of reference organisms have influence on the performance of ION, some experiments are carried out. The experimental results show that using all available reference organisms can improve the performance of ION. At the last part of the paper, we compare the prediction performance of ION with that of other seven centrality methods (DC, BC, CC, SC, EC, IC and NC), based on proteins from *E. coli* K-12 (*E*. coli). Results confirm that ION gets better performance on prediction of essential proteins in *E. coli* than the seven centrality methods.

## Methods

Proteins in cells are not independent. They interact with each other and construct PPI networks. A PPI network can be represented by an undirected graph *G=(V, E)*, where *V* is the set of nodes (proteins) and *E* is the set of edges (binary interactions). Many prediction methods select essential proteins in a PPI network by ranking them according to some criteria. The outcome is an ordered list of proteins, such that the proteins near the top of the list are most likely to be essential.

ION is developed to compute the ranking scores of proteins. It initializes the ranking scores of proteins with their orthologous scores. For sake of modularity of essential proteins, the edges connecting proteins are associated with weights. Finally, the ranking scores are computed by considering the orthologous scores, the neighbors’ features and the connections of proteins. Since the ranking scores of proteins relate to the scores of their neighbors, an iteration process is proposed.

### Experimental data and analysis

The computational analysis is performed using the PPI network from *S*. cerevisiae (Bakers’ Yeast), because both its PPI and gene essentiality data are the most complete and reliable among various species. The PPI data of *S*. cerevisiae is downloaded from DIP database [37] updated to Oct.10, 2010, without self-interactions and repeated interactions. There are total of 5093 proteins and 24743 interactions. The list of 1285 essential proteins is integrated from the following databases: MIPS [38], SGD [39], DEG [40], and SGDP [41]. Among the 1285 essential proteins, 1167 essential proteins present in the PPI network. In our study, these 1167 proteins are considered as essential proteins while other 3926(=5093-1167) proteins are nonessential proteins. Information on orthologous proteins is taken from Version 7 of the InParanoid database (an ortholog database) which contains a collection of pairwise comparisons between 100 whole genomes (99 eukaryotes and 1 prokaryote) constructed by the INPARANIOD program. In our study, only the proteins in seed orthologous sequence pairs of each cluster generated by INPARANIOD are chosen as orthologous proteins, because they have the best match between two organisms and stand for the high homology. The UNIPORT flat file is used to match DIP entries with the Ensembl Gene IDs used by InParanoid to index yeast genes.

**Figure 1:**
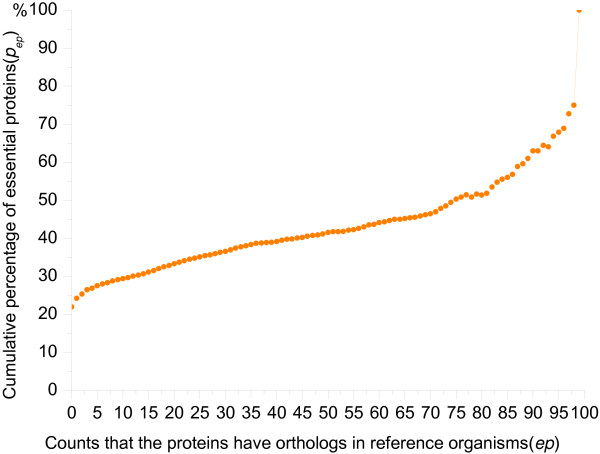
**Cumulative percentage of essential proteins in orthologs sets.** Figure1 shows the percentages of essential proteins out of the proteins that have orthologs in 99 reference organisms not less than number *ep* of times. It illustrates the relationship between the essentiality properties of proteins and the number of orthologs that they have in reference organisms.

In order to get the relationship between essentiality and orthologous properties of proteins, we check the yeast proteins if they have orthologs in 99 reference organisms ranging from *H*.sapiens to *E. coli*. As a result, 4511 proteins (present in the yeast PPI network) are labeled to have orthologs in at least one of 99 reference organisms. Furthermore, 1118 out of 1167 known essential proteins are included in these 4511 proteins. It means that 96% (1118/1167) of essential proteins in the PPI network are evolutionarily conserved. For further analysis, *Pep* is used to describe the percentage of essential proteins out of all proteins that occur in orthologous seed pairs not less than number *ep* of times, here *ep* ranges from 1 to 99. Figure 1 outlines the data.

As Figure 1 indicated, with the increase of *ep* the value of *Pep* rises accordingly. For instance, when proteins have orthologs in at least 80 organisms, the percentage of essential proteins in them is about 51%. Note that one protein having orthologs in all 99 organisms is an essential protein. However, from the proteins that don’t have orthologs in any one of 99 organisms, 22 percent are essential proteins, near to random probability [42]. Consequently, we can conclude that the more frequently the yeast proteins appear in orthologous seed pairs with respect to reference organisms, the more possible they are essential. This just confirms that essential proteins are relatively conserved. Therefore, in this study we will explore the orthologous properties to predict essential proteins.

### Orthologous score

The first step of ION is to assign an orthologous score to each protein in a PPI network. To formally define the orthologous score, the following variables are introduced.

Given a PPI network represented as a graph *G=(V, E)*, *N* denotes the number of node in set *V*.

Let *S* be the set of reference organisms which is used to get orthologous information of node *V*. *s* denotes its element. *|S|* denotes the number of its elements.

Let *X*_*s*_ be a subset of node *V*. Its element has orthologs in organism *s*.

Let *o(i)* be the number of times that node *v*_*i*_ has orthologs in reference organisms, where *v*_*i*_ ∈ *V* (i=1,…,*N*).

$o\left(i\right)=\sum_{m\in S}T_{i}$ where $T_{i}=\{\begin{array}{ccc} 1 & if & v_{i}\in X_{m}\\ 0 & & otherwise \end{array}$

Then the orthologous score *d(i)* of node *v*_*i*_ is defined as

(1) $$d(i)=\frac{o(i)}{\underset{i\in N}{Max(o(i))}}$$

According to the definition, for proteins that have orthologs in all organisms included in set *S*, their orthologous scores are 1. On the contrary, for proteins that don’t have orthologs in any one of organisms in set S, their orthologous scores are 0.

For proteins in yeast PPI network, following steps are taken to assign orthologous scores to them.

Assignment of orthologous score

Step1. Let set *S* include 99 organisms ranging from *H.sapiens* to *E. coli*.

Step2. Retrieve a list of set *X*_*s*_ from the InParanoid database with respect to species *s*, where *s* ∈ *S*.

Step4. For each $v_{i}\in V$, compute *o(i).*

Step5. For each $v_{i}\in V$, compute *d(i)*.

### Weighting edges

Although it is believed that an essential protein tends to have a high correlation with its connectivity, there are many nonessential proteins having high connections while a certain ratio of essential proteins having low connections in reality. Hart et al. [43] indicated that essential proteins strongly cluster together. The essentiality is the product of protein complexes rather than individual proteins. This indicates the modular nature of essential proteins. The connections between the nodes in a complex are denser than connections with the rest in networks. To describe how close two proteins are, the edge-clustering coefficient is introduced. It is widely used to identify the modularity of networks [44,45]. The edges with the higher clustering coefficient are more probably involved in the community structure in networks. Therefore, a node has a high probability to be essential if it possesses more adjacent edges with a higher edge-clustering coefficient. Those proteins with a high degree are nonessential proteins because the edge-clustering coefficients of their adjacent edges are relatively low. By contrast, those proteins with the low connectivity are essential because the edge-clustering coefficients of their adjacent edges are relatively high. Based on the edge-clustering coefficient, Wang et al. [21] propose method NC to predict essential proteins. This method gives proteins scores according to the sum of edge-clustering coefficients of their adjacent edges and outperforms other six centrality measures (DC, BC, CC, SC, EC and IC) in predicting essential proteins.

In this work, we associate a weight with each edge in terms of its edge-clustering coefficient. It is defined as the number of triangles to which a given edge belongs, divided by the number of triangles that might potentially include it. Mathematically it can be expressed as follows:

(2) ECCi,j=zi,jminki−1,kj−1

where *Z*_*i, j*_ is the number of triangles built on *edge(v*_*i*_*,v*_*j*_*)*. *k*_*i*_ and *k*_*j*_ are the degrees of nodes *v*_*i*_ and *v*_*j*_, respectively. *min(k*_*i*_*-1, k*_*j*_*-1)* is actually the maximal possible number of triangles that might potentially include the *edge* (*v*_*i*_*,v*_*j*_). In a weighted network, the correlations between proteins and their neighbors are thus not identical. Furthermore, the essentiality of a protein tends to depend on that of its neighbors if the edges connecting the protein and its neighbors have high edge-clustering coefficients. Note that differently from NC, ION predicts essential proteins taking into account not only the connections between proteins but also the orthologous properties of proteins and the features of their neighbors.

### Computation of ranking scores

After computing the edge-clustering coefficient for each edge, an adjacency matrix is constructed to represent the connections between proteins. Its element is the normalized edgeclustering coefficient of each edge.

Let H be an N×N adjacency matrix of the graph *G=(V, E)*. Its element *h(i, j)* is defined as follows.

(3) $$h\left(i,j\right)=\{\begin{array}{ccc} Norm_{i}\left(ECC\left(i,j\right)\right)=\frac{ECC\left(i,j\right)}{\sum_{w\in Ne\left(i\right)}ECC\left(i,w\right)} & if & \sum_{w\in Ne\left(i\right)}ECC\left(i,w\right)>0\\ 0 & & otherwise \end{array}$$

where *Ne(i)* is the set of neighbors of node *v*_*i*_ and *ECC(i, j)* denotes the edge-clustering coefficient of *edge(v*_*i*_*,v*_*j*_*)*. Therefore, for each row *i* of matrix *H*, either $\sum_{j\in Ne\left(i\right)}h\left(i,j\right)=1$or $\sum_{j\in Ne\left(i\right)}h\left(i,j\right)=0$.

Let *pr(i)* be the ranking score of node *v*_*i*_. Define the neighbor-induced score as $\sum_{j\in Ne\left(i\right)}h\left(i,j\right)pr\left(j\right)$. Then for each protein in the PPI network, its ranking score can be computed as follows.

(4) $$pr\left(i\right)=\left(1-\alpha \right)d\left(i\right)+\alpha \sum_{j\in Ne\left(i\right)}h\left(i,j\right)pr\left(j\right)$$

From the above definition, the ranking score of a protein is viewed as a linear combination of its orthologous property score and the neighbor-induced score. The parameter α (0≤α<1) is used to adjust the weight of two scores in the ranking score. As the value of α is equal to 0, the ranking score only depends on the orthologous properties of proteins. If the value of α is between 0 and 1, ranking scores are computed based on both the orthologous properties of proteins and their neighbor’s features. In Equation (4), for a node *i*, *pr(j)* presents the score of its neighbor *j*. Value in element *h(i, j)* depends on the edge-clustering coefficient of *edge(v*_*i*_*, v*_*j*_*)*. The ranking score of node *i* has correlation with all of its neighbors which are not treated equally. The correlations between node *i* and its neighbors depend on the values of elements in matrix *H*.

It can be seen that Equation (4) is a coupled linear system in unknown *pr(i)*. As the number of unknowns is very large, it is impossible to analytically solve this system. To numerically solve Equation (4), we rewrite Equation (4) in the matrix–vector format as follows

(5) $$pr=\left(1-\alpha \right)d+aH*pr$$

where pr=(pr(1),….pr(N)) and d=(d (1),….,d(N)). In this study, we adopt the Jacobi iterative procedure to numerically solve Equation (5) as follows

(6) $$pr^{t+1}=\left(1-\alpha \right)d+aH*pr^{t}$$

*t* (=0,1,2,…) represents the iteration steps.

### Algorithm

As described in ION algorithm below, based on input data each protein is initialized with an orthologous score in terms of its orthologous property. Then, for sake of modularity of essential proteins, the edge-clustering coefficient of each edge is computed. After that, the ranking scores of proteins are calculated by taking consideration of their orthologous scores and their neighbor-induced scores as in Equation (4). A dumping factor α is used to adjust the contribution of their orthologous scores and their neighbor-induced scores. The algorithm is convergent. The number of iteration depends on parameters α and ε. The proof of convergence and the discussion of the effect of related parameters on the algorithm convergence can be found in Additional file 1.

ION algorithm

**Input:** A PPI network represented as Graph G=(*V*, *E*), orthologs data sets between Yeast and 99 other organisms (ranging from *H.sapiens to E. coli*), stopping error ε, parameter α, parameter *K*.

**Output:** Top *K* percent of proteins sorted by *pr* in descending order

Step1: Calculate each protein orthologous score by Equation (1)

Step2: Compute the edge-clustering coefficient of each edge by Equation (2)

Step3: Construct matrix *H* by Equation (3)

Step4: Initialize pr with pr^0^=d, let t=0

Step5: Compute pr^t+1^ by Equation (6), let t=t+1

Step6: Repeat step 5 until

(7) $${||pr^{t}-pr^{t-1}||}_{1}\leq \varepsilon$$

Step7: Sort proteins by the converged value of *pr* in the descending order.

Step8: Output top *K* percent of sorted proteins.

## Results and discussion

In order to evaluate the essentiality of proteins in PPI networks, they are ranked in the descending order based on their ranking scores computed by ION in Section 2,5 as well as eight other existing centrality methods (DC, BC, CC, SC, EC, IC, NC and PeC). After that, top 1%, 5%, 10%, 15%, 20% and 25% of the ranked proteins are selected as candidates for essential proteins. According to the list of known essential proteins, the number of true essential proteins is used to judge the performance of each method. This evaluation measure has been widely used in earlier research procedures [21-23,42],].

In this section, we first discuss the effect of parameter*α*on the performance of ION. Then we compare ION with eight other existing centrality methods. After that, the results of ION and eight other existing centrality methods are analyzed in details. Furthermore, we discuss whether the number of reference organisms influences on the prediction performance of ION. Finally, the prediction performance of ION is tested on a protein dataset of *E. coli*.

### Effects of parameter α

**Figure 2:**
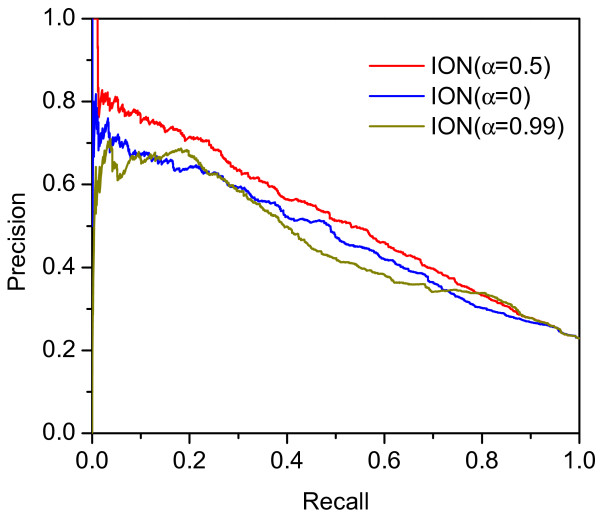
**PR curves of ION when α is set as 0, 0.5 and 0.99.** In ION, the ranking score of a protein which stands for its essentiality in PPI network is viewed as a linear combination of its orthologous score and the neighbor-induced score. The parameter α is used to adjust the weight of two score in the ranking score. As the value of α is equal to 0, the ranking scores only depends on the orthologous properties of proteins. As the value of α is equal to 0.99, the ranking score almost only depends the neighbor’s information. When α is set as other values ranging from 0.1 to 0.9, such as 0.5, the prediction is implemented by integrating the proteins’ orthologous property with their neighbor’s property in the PPI network. The Figure shows precision-recall (PR) curves of ION when α is set as 0, 0.5 and 0.99, respectively.

In ION, the ranking scores of proteins are changed with different values of α. To study the effect of parameter α on performance of ION, we evaluate the prediction accuracy by setting different values of α, ranging from 0 to 0.99. The detailed results are listed in Table 1. Here, the parameter *K* is from top 1% to top 25%. The prediction accuracy is measured in terms of the percentage of true essential proteins in candidates. Moreover, to display the overall performance of ION when α is set as 0, 0.5 and 0.99, we plot their PR curves in Figure 2.

**Table 1:** Effect of parameter α on the performance of ION

***K***	**0**	**0.1**	**0.2**	**0.3**	**0.4**	**0.5**	**0.6**	**0.7**	**0.8**	**0.9**	**0.99**
1%	0.75	0.84	0.78	0.78	0.78	0.80	0.80	0.78	0.80	0.76	0.69
5%	0.65	0.70	0.72	0.73	0.73	0.74	0.74	0.74	0.73	0.71	0.67
10%	0.62	0.66	0.65	0.66	0.65	0.65	0.64	0.64	0.63	0.61	0.61
15%	0.56	0.58	0.59	0.58	0.58	0.58	0.58	0.58	0.57	0.57	0.54
20%	0.51	0.52	0.52	0.53	0.55	0.54	0.54	0.53	0.52	0.51	0.48
25%	0.47	0.47	0.48	0.49	0.50	0.50	0.48	0.48	0.48	0.46	0.43

The table shows the effect of parameter α on the performance of ION. Column ‘*K*’ represents top *K* percent of ranked proteins. Column 2 to 11 represents prediction accuracy of ION in each top percentage of ranked proteins by setting different values of α, ranging from 0 to 0.99.

Both Table 1 and Figure 2 shows that for α=0 the prediction only considers orthologous properties of proteins. Its results are comparable with those for α=0.99, where the prediction almost only considers the neighbor’s information. Furthermore, the prediction performance when α is either 0 or 0.99 is poorer than when α is set as other values ranging from 0.1 to 0.9, which means that the prediction performance by integrating the proteins’ orthologous property with their neighbor’s property in the PPI network is better than that by using only either property. With each top percentage (1%, 5%, 10%, 15%, 20%, or 25%), the value of parameter α ranging from 0.1 to 0.9 has some effects on the prediction performance of ION but it is not crucial. Furthermore their medium values are 0.784, 0.729, 0.649, 0.581, 0.530 and 0.483, respectively, which are near to the corresponding prediction accuracy when α is 0.5. As a result, we think the optimum α value is 0.5.

### Comparison with eight centrality methods

**Figure 3:**
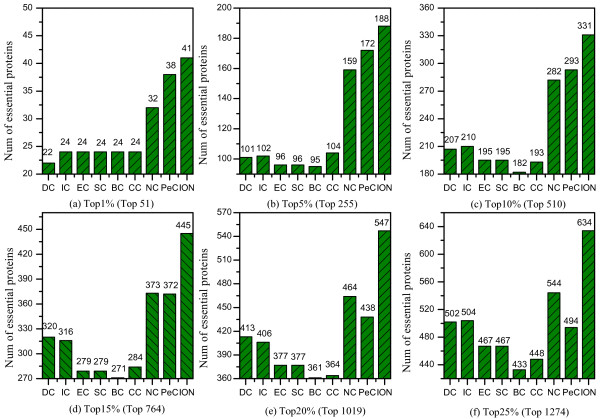
**Number of essential proteins identified by ION and eight other existing centrality methods.** The proteins in PPI network are ranked in the descending order based on their ranking scores computed by ION, Degree Centrality (DC), Betweenness Centrality (BC), Closeness Centrality (CC), Subgraph Centrality (SC), Eigenvector Centrality (EC), Information Centrality (IC), Edge Clustering Coefficient Centrality (NC), and centrality based on edge clustering coefficient and Pearson correlation coefficient (PeC). Then, top 1%, 5%, 10%, 15%, 20% and 25% of the ranked proteins are selected as candidates for essential proteins. According to the list of known essential proteins, the number of true essential proteins is used to judge the performance of each method. The figure shows the number of true essential proteins identified by each method in each top percentage of ranked proteins. Since the total number of ranked proteins is 5093. The number of proteins ranked in top 1% is about 51(=5093*1%). The digits in brackets denote the number of proteins ranked in each top percentage.

To evaluate the performance of ION, we compare the number of essential proteins identified by ION (α=0.5) and eight other existing centrality methods, when selecting various top percentages of ranked proteins as candidates for essential proteins. Figure 3 shows the comparison of results.

As illustrated in Figure 3, the prediction performance of ION has a significant improvement. By selecting top 1% of proteins, ION obtains a prediction accuracy of 80%. With top 25% of proteins selected, ION can detect 50% of true essential proteins. Compared with NC which has the best performance among seven existing centrality methods (DC, BC, CC, SC, EC, IC and NC), in each top percentage (1%, 5%, 10%, 15%, 20%, and 25%), The prediction accuracy of ION is improved by 28.13%, 18.24%, 17.36%, 19.30%, 17.89%, 16.54%, respectively. While compared with DC which is widely used in essential protein prediction, ION shows remarkable performance. Particularly, for top 1% of ranked proteins, ION is capable of identifying almost twice as many essential proteins as DC. As top 5% and top 10% of proteins selected, the prediction performance of ION achieves 86% and 60% improvement, compared with DC, respectively. Compared with PeC which identifies essential proteins by integrating gene expression data with PPI network data, ION also outperforms it. Especially, with more candidate proteins selected, the advantage of ION in the prediction of essential proteins becomes increasingly obvious.

### Validated by precision-recall curves

**Figure 4:**
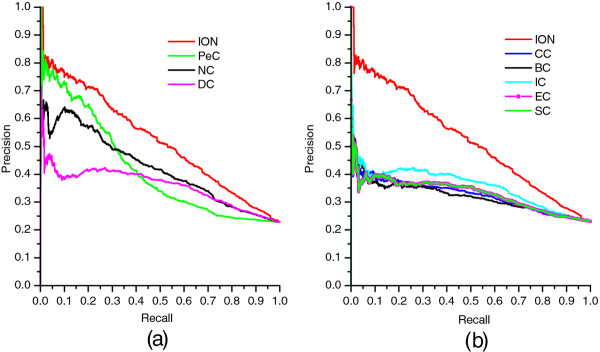
**PR curves of ION and eight other existing centrality methods.** The proteins ranked in top *K* (cut-off value) by each method (ION, DC, BC, CC, SC, EC, IC, NC and PeC) are selected as candidate essential proteins (positive data set) and the remaining proteins in PPI network are regarded as candidate nonessential proteins (negative data set). With different values of *K* selected, the values of precision and recall are computed for each method. The values of precision and recall are plotted in PR curves with different cut-off values. **(a)** shows the PR curves of ION, PeC, NC and DC. **(b)** shows the PR curves of ION and two global centrality methods: BC and CC, and other tree centrality methods: IC, EC and SC.

Moreover, we also employ precision-recall (PR) curves and the corresponding areas under the PR curve (AUC) values to evaluate the overall performance of each method. At the beginning, the proteins in PPI networks are ranked in the descending order according to the ranking scores computed by each method. After that, the top *K* proteins are selected as candidate essential proteins (positive data set), then the remaining proteins in PPI networks are regarded as candidate nonessential proteins (negative data set). The cut-off values of *K* range from 1 to 5093. With different values of *K* selected, the values of precision and recall are computed for each method, respectively. Then, the values of precision and recall are plotted in PR curves with different cut-off values. The experimental results are illustrated in Figure 4. Figure 4 (a) shows the PR curves of ION, PeC, NC and DC. Figure 4 (b) shows the PR curves of ION and two global centrality methods: BC and CC, and other tree centrality methods: IC, EC, SC. Note that the PR curves of EC and SC are undistinguishable in Figure 4(b). From Figure 4, we can see that the PR of ION is clearly above those of all other methods.

### Validated by jackknife methodology

**Figure 5:**
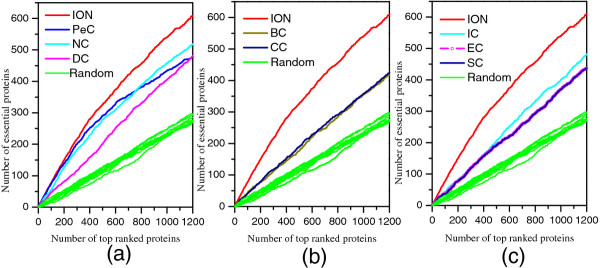
**Jackknife curves of ION and eight other existing centrality methods.** The x-axis represents the proteins in PPI network ranked by ION and eight other existing centrality methods, ranked from left to right as strongest to weakest prediction of essentiality. The Y-axis is the cumulative count of essential proteins encountered moving left to right through the ranked. The areas under the curve for ION and the eight other existing centrality methods are used to compare their prediction performance. In addition, the 10 random assortments are also plotted for comparison. **(a)** shows the comparison results of ION, PeC, NC and DC. **(b)** shows the comparison results of ION and two global centrality methods: BC and CC. **(c)** shows the comparison results of ION and other three centrality methods: IC, EC and SC.

For further comparison, the jackknife methodology [46] is used to test the prediction performance of ION and the eight other existing centrality methods. The experimental results are described in Figure 5, where the x-axis from left to right represents the proteins in PPI networks ranked in the descending order according to their ranking scores computed by corresponding methods while the Y-axis is the cumulative count of essential proteins with respect to ranked proteins moving left to right. The areas under the curve for ION and the eight other existing centrality methods are used to compare their prediction performance. In addition, the 10 random assortments are also plotted for comparison. Figure 5 (a) shows the comparison result of ION, PeC, NC, DC. From this figure, ION has consistently excelled PeC which identifies essential proteins by integrating gene expression data with PPI data. Figure 5 (b) shows the comparison result of ION and two global centrality methods: BC and CC. Figure 5 (c) shows the comparison result of ION and other three centrality methods: IC, EC, SC. Compared with the seven centrality methods, ION also outperforms them. Moreover, all of the nine methods achieve better prediction performance than the randomized sorting. 

### Analysis of the differences between ION and the eight centrality methods

To understand why and how ION gets better results than the eight other existing centrality methods, firstly we compare them in identifying essential proteins with low-connectivity (degrees less than 10). A significantly proportion (76% on average) of proteins in the yeast PPI datasets are of low-connectivity. 58% of essential proteins in known essential proteins list also have low connectivity in the PPI networks. However, the eight other existing centrality methods are connectivity-based, which results in proteins with low-connectivity are largely missed out. ION implements the prediction of proteins essentiality depending on orthologous properties of proteins and the features of their neighbors, which can compensate the shortcomings of centrality methods.

As indicated in the top part of Table 2, for each top percentage of proteins, ION finds more essential proteins with low-connectivity than eight other existing centrality methods. From Table 2, the six centrality methods (DC, IC, EC, SC, BC and CC) can barely find essential proteins with low-connectivity in up to top 10% of proteins while ION discovers many of these. With top 25% of proteins selected, the number of essential proteins with low-connectivity found by ION is almost five times of the average number found by the six centrality methods. Although NC and PeC find some essential proteins with low-connectivity, ION is able to find essential proteins with low-connectivity about 60% and 30% more than NC and PeC, respectively, as top 25% of proteins are selected. The bottom part of Table 2 describes that ION also finds a large number of essential proteins with high connectivity (degree >10). Up to top 10% of proteins, ION detects more essential proteins with high-connectivity than the six centrality methods (DC, IC, EC, SC, BC and CC). In top 25% of proteins, ION still outperforms the methods BC in finding essential proteins with high-connectivity, while that performance of SC, EC, CC and NC only slightly better than ION. Compared with PeC, with more candidate proteins selected, the performance of ION in identifying essential proteins with high-connectivity surpasses that of PeC. In summary, ION achieves comparable performance in finding essential proteins with high-connectivity with some of the eight other existing centrality methods. However, ION can also discover a large number of essential proteins with low-connectivity ignored by the eight other existing centrality methods. This can explain the great performance of ION in the prediction of essential proteins.

**Table 2:** Number of essential proteins with low-connectivity or high-connectivity identified by ION and eight other existing centrality methods

	***K***	**DC**	**IC**	**EC**	**SC**	**BC**	**CC**	**NC**	**PeC**	**ION**
deg<=10	1%	0	0	0	0	0	0	0	0	17
	5%	0	0	0	0	0	0	3	40	66
	10%	0	0	0	0	1	0	27	84	108
	15%	0	0	8	8	18	7	66	117	146
	20%	0	0	28	28	41	20	101	153	188
	25%	11	20	73	73	76	55	156	192	253
deg > 10	1%	22	24	24	24	24	24	32	39	24
	5%	101	102	96	96	95	104	156	133	122
	10%	207	210	195	195	181	193	255	209	223
	15%	320	316	271	271	253	277	307	255	299
	20%	413	406	349	349	320	344	363	285	359
	25%	491	484	394	394	357	393	388	302	381

The top part and the bottom part of this table are the number of essential proteins with low-connectivity (degree < =10) and high-connectivity (degree > 10) identified by different methods, when selecting top *K* percent of proteins (top 1%, 5%, 10%, 15%, 20%, 25%).

**Figure 6:**
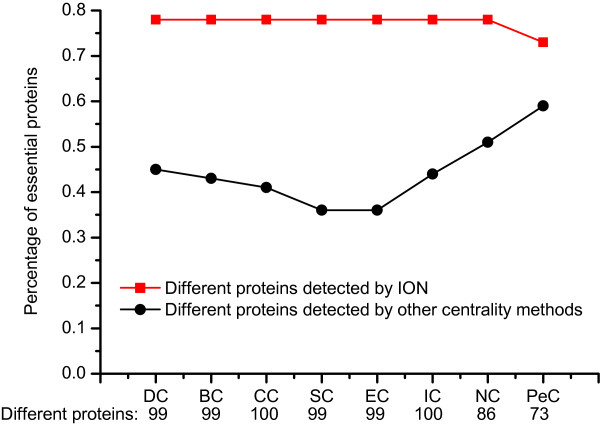
**Percentages of different essential proteins resulted by ION and eight other existing centrality methods.** Different proteins between two prediction methods are the proteins predicted by one method while neglected by the other method. The figure shows the percentages of the essential proteins in the different proteins between ION and eight other existing centrality methods (DC, BC, CC, SC, EC, IC, NC and PeC), respectively.

Secondly, we compare proteins ranked in top 100 by each method (DC, IC, EC, SC, BC, CC, NC, PeC and ION) to view how many overlap and different proteins are identified by these methods. In Table 3, |ION ∩ Mi | denotes the number of proteins detected by both ION and one of the eight other existing centrality methods Mi. {Mi–ION} represents the set of proteins detected by Mi ignored by ION. |Mi–ION| is the number of proteins in set {Mi–ION} .As described in Table 3, there exist huge differences between the proteins identified by ION and Mi. Taking DC, IC, EC, SC, BC and CC for example, there are almost no common proteins identified by both ION and them. For NC and PeC, there are only few proteins identified by both ION and them. These results show that ION is a special method compared with the other methods. For further analysis, we compare the percentages of different essential proteins resulted by ION and by the eight other existing centrality methods. As shown in Figure 6, ION can detect more different essential proteins than these methods. Compared with PeC, there are 73 different proteins detected by ION. 53 out 73(about 73%) of these proteins are essential. By contrast, there are only 54% of different proteins detected by PeC while ignored by ION are essential proteins. In fact, for the top 100 of proteins, ION can detect 52 different essential proteins which can’t be detected by anyone of the eight other existing centrality methods (see Additional file 2). Additionally, we also find that more than 50% of nonessential proteins in top 100 ranked by DC, IC, EC, SC, BC and CC possess low ranking scores (less than 0.55) computed by ION. As we can see from Table 3, about 70% of nonessential proteins in the result of NC have low ION ranking scores. More over, among the top 100 of proteins predicted by PeC, there also exist about 46% of nonessential proteins with low ION ranking scores. This means that ION can exclude many nonessential proteins which can’t be ignored by the other methods. Since ION can not only detect more essential proteins ignored by the eight other existing centrality methods but also exclude a large number of nonessential proteins which can’t be ignored by these methods, it is not surprise that ION has high performance in the prediction of essential proteins.

**Table 3:** Overlap and different proteins identified by ION and eight other existing centrality methods

**Centrality measures (Mi)**	**|ION∩Mi|**	**|Mi − ION|**	**nonessential proteins in {Mi − ION}**	**non-essential proteins percentage in {Mi − ION} with low ION value**
Degree Centrality (DC)	1	99	54	59.26%
Betweenness Centrality (BC)	1	99	56	57.14%
Closeness Centrality (CC)	0	100	59	55.93%
Subgraph Centrality(SC)	1	99	63	52.38%
Eigenvector Centrality(EC)	1	99	63	52.38%
Information Centrality(IC)	0	100	56	55.36%
Edge Clustering Coefficient Centrality (NC)	14	86	42	73.81%
PCC and ECC centrality(PeC)	27	73	24	45.83%

This table shows the common and the difference between ION and the eight other existing centrality methods (DC, BC, CC, SC, EC, IC, NC and PeC) when predicting top 100 proteins. |ION ∩ Mi | denotes the number of proteins identified by both ION and one of the eight other existing centrality methods Mi. {Mi − ION} represents the set of proteins detected by Mi while ignored by ION. |Mi − ION| is the number of proteins in set {Mi − ION}. The last column describes the percentages of different nonessential proteins with low ION scores (less than 0.55) in top 100 proteins.

### Modularity, orthology and essentiality of the proteins ranked by ION

**Figure 7:**
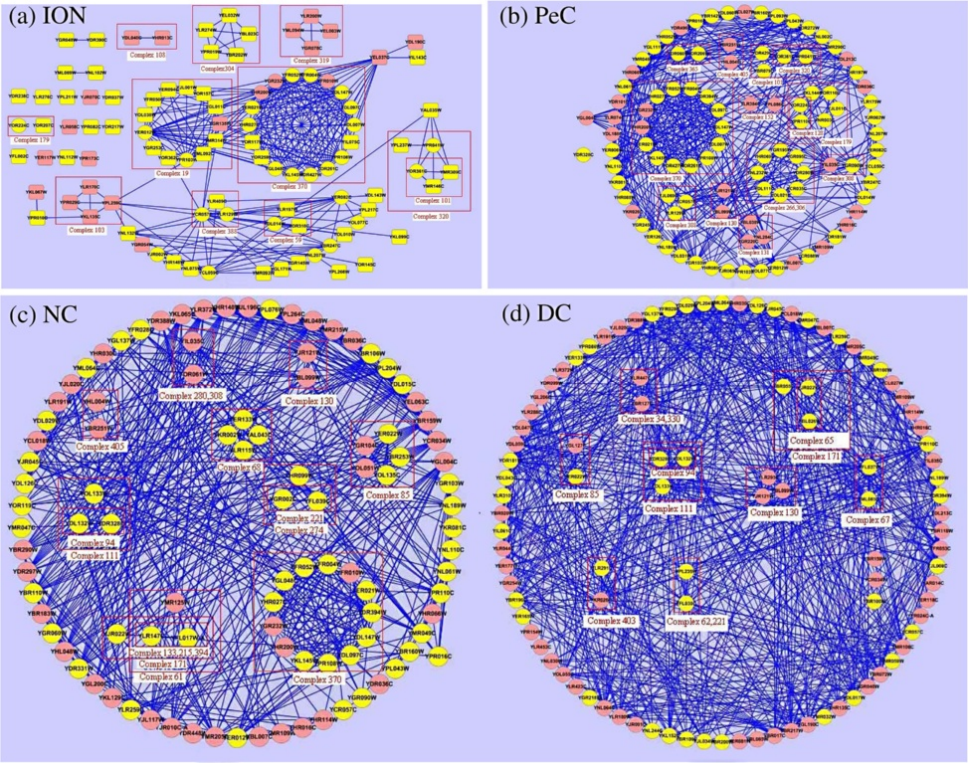
**Proteins ranked in top 100 by ION, PeC, NC and DC and the complexes they belong to.** The figure shows the proteins ranked in top 100 by ION, PeC, NC and DC, and the networks constructed by these proteins. The proteins included in a red square belong to a common complex. The yellow nodes denote true essential proteins. In (a), the nodes with the shape of round rectangle represent the different proteins detected by ION while ignored by all of the eight other existing centrality methods.

Since ION is designed by considering the orthology, connectivity, modularity and neighbor dependency of proteins, the proteins with high ranking scores computed by ION should be conserved, essential and connect with each other. To verify this hypothesis, we select a list of proteins ranked in top 100 by ION, NC, PeC and DC, respectively. According to the known 408 manually annotated complexes [47], proteins in each list are annotated with the index of complexes which they belong to. The interaction networks of these proteins are visualized by using the software CYTOSCAPE [48]. Figure 7 shows these networks. The proteins included in a red square belong to a common complex. The yellow nodes denote true essential proteins. Table 4 lists the statistic information of these proteins. More detailed information about these proteins and the corresponding complexes is listed in the Additional file 3. From Figure 7 and Table 4, compared with PeC, NC and DC,we can clearly see that more true essential proteins are detected by ION, but also more of these proteins ranked in top 100 by ION belong to the complexes with certain biological functions. The average count that the proteins ranked in top 100 by ION have orthologs in reference organisms is about 93, 78 out of 100 these proteins are essential and 72 out of 100 these proteins belong to the complexes. By contrast, the average count that the proteins ranked in top 100 by PeC has orthologs in reference organisms is about 78, 74 out of 100 these proteins are essential and 57 out of 100 these proteins belong to the complexes. Additionally, as indicated in Table 4, the sub-complexes containing the proteins ranked in top 100 by ION have higher interaction rate with known complexes than that containing the proteins ranked by other methods. For example, there 18 proteins ranked in top 100 by ION belong to complex 370. The complex 370 is 19/22 S regulator and its GO term is GO: 0008541 with function of proteasome regulatory particle, lid subcomplex. For PeC and NC, there are only 14 proteins and 13 proteins in top 100 proteins ranked by them belong to the complex 370, respectively. 

**Table 4:** Information of proteins ranked in top 100 by ION, PeC, NC and DC

**method**	**Number of essential proteins**	**Number of proteins belonging to complex**	**Average number of orthologs**	**average interaction rate with known complex**
ION	78	72	93	0.39
PeC	74	57	78	0.37
NC	55	59	65	0.32
DC	46	53	62	0.33

This table shows the statistic information of proteins ranked in top 100 by ION, PeC, NC and DC. Column “Number of essential proteins” presents the number of essential proteins in the proteins ranked in top 100 by corresponding methods. Column “Number of proteins belonging to complex” presents how many proteins ranked in top 100 by corresponding methods belong to a least one known complex. Column “Average number of orthologs” presents the average number of orthologs that the proteins ranked in top 100 by corresponding methods have in reference organisms. Column “average interaction rate with known complex” presents the average interaction rate between the sub-complexes including these proteins and known complexes.

### Discussions on the orthologous score

**Figure 8:**
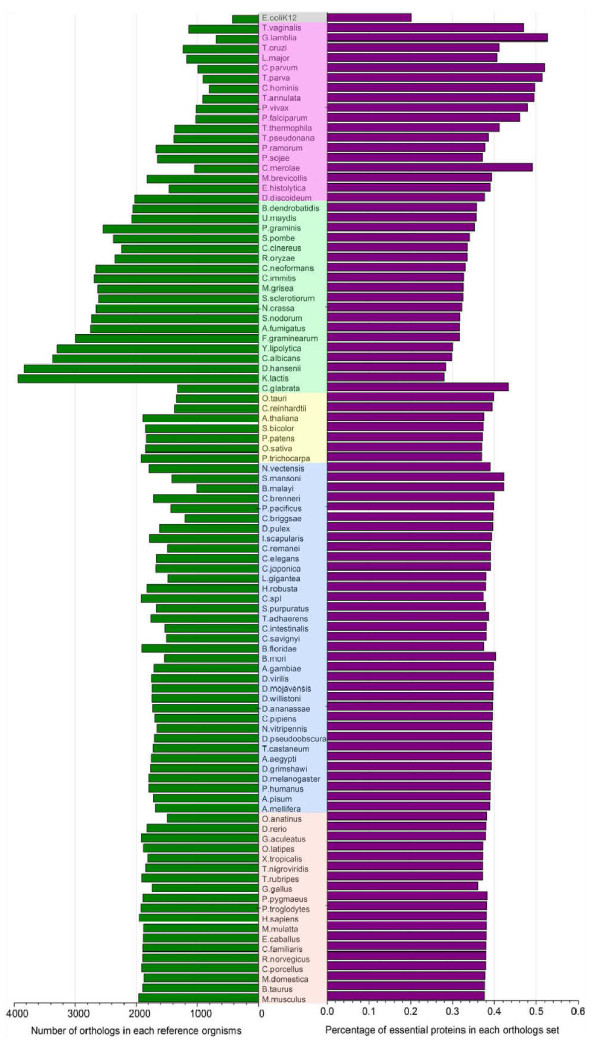
**Number of yeast orthologs in each reference organism and percentage of essential proteins in each ortholog set.** The figure lists 99 reference organisms. These organisms are ordered by their phylum and the decreasing percentage of essential proteins out of their yeast orthologs (red: vertebrate, blue: invertebrate, yellow: plant, green: fungi, purple: protist, prey: prokaryote). The number of yeast proteins which have orthologs in each reference organism is shown in the left part of the figure. The percentage of essential proteins in each ortholog set is shown in the right part of the figure.

We assign orthologous scores to yeast proteins based on the counts they have orthologs in 99 organisms. The orthologous data can be conveniently obtained from the Inparanoid database. How about the performance of ION if we select a small number of reference organisms? Hence, according to known essential protein data in yeast, we first calculate how many proteins have orthologs in each of the 99 reference organisms and then analyze the percentages of essential proteins in each ortholog set. With respect to NCBI Taxonomy common tree, the 99 organisms are divided into six groups. They include 19 vertebrates, 35 invertebrates, 7 plants, 19 fungus, 18 protists and 1 prokaryote (*E. coli*). Figure 8 illustrates the detailed information.

The average percentage of essential proteins in each group is vertebrate: 37.69%, invertebrate: 39.24%, plant: 38.36%, fungi: 32.57%, protist: 44.43% and prokaryote: 20.10%. For all organisms except *E. coli*, the percentage of essential protein is higher than random probability. This is in agreement with the finding that essential proteins are more conserved than nonessential proteins. It is not surprise that the percentage of essential proteins in the proteins that have orthologs in *E. coli* is the lowest because of distant relation between yeast and *E. coli*. Another interesting discovery is that even if there are a small number of proteins having orthologs in protistan organisms, the ratio of essential proteins to those proteins is high. It may be the protists are old eukaryotic organisms. By contrast, although a large number of proteins have orthologs in fungal organisms which have close evolutionary distances with yeast, they generate a relatively low ratio of essential proteins. In spite of the big difference among organisms in group vertebrate, invertebrate and plant, they generate general similar percentage of essential proteins. All of these findings can provide us some helpful information about selecting appropriate reference organisms.

**Figure 9:**
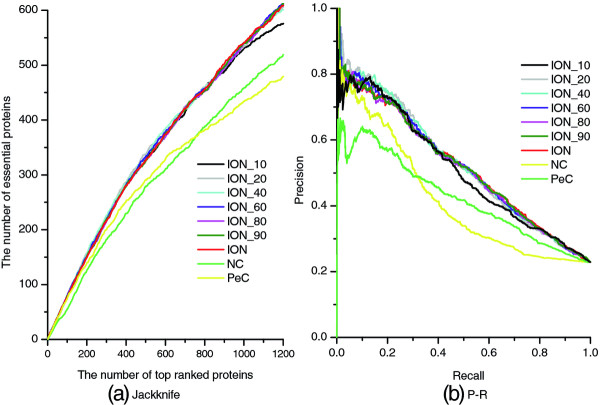
**Jackknife curves and PR curves of NC, PeC and different ION results.** The prediction performance of ION with respect to different number of reference organisms are validated by the jackknife method and the PR method, respectively. All of those results are also compared with both NC and PeC by using the jackknife method and the PR method.

To check the influence of referent organisms on prediction performance, according to Taxonomy common tree, we assign orthologous scores to proteins by selecting 10, 20, 40, 60, 80, 90 organisms as reference (see Additional file 4), respectively. The prediction results are correspondingly named by ION_10, ION_20, ION_40, ION_60, ION_80 and ION_90. Table 5 shows the number of true essential proteins in top 1%, 5%, 10%, 15%, 20% and 25% of these results. Furthermore, these results are also validated by PR curve and jackknife curve, which are illustrated in Figure 9. It can be seen from Table 5 and Figure 9 that no matter how many organisms are selected as references, the prediction accuracy of ION surpasses that of both NC and PeC. In general, the more reference organisms are used, the better prediction performance of ION can be achieved. However, when selecting more than 10 organisms as references, the difference of these results is not obvious.

**Table 5:** Number of essential proteins identified by ION with respect to different number of reference organisms

**Results**	**1%**	**5%**	**10%**	**15%**	**20%**	**25%**
ION_10	39	192	335	442	534	601
ION_20	42	195	344	447	540	627
ION_40	42	197	338	443	549	627
ION_60	40	190	337	448	543	631
ION_80	40	182	331	442	542	630
ION_90	41	185	331	445	544	637
ION	41	188	331	445	547	634

Row ‘ION_10’, ‘ION_20’, ‘ION_40’, ‘ION_60’, ‘ION_80’, ‘ION_90’ and ‘ION’ show the prediction results of ION in each percentage of proteins when selecting 10, 20, 40, 60, 80, 90 and 99 organisms as reference, respectively.

### Prediction performance of ION based on protein data from *E. coli*

To further evaluate the performance of ION, we perform the prediction of essential proteins in *E. coli*. The PPI data of *E. coli* is also downloaded from DIP database updated to Oct.10, 2010. There are total of 2727 proteins and 11803 interactions. The self-interactions and repeated interactions are ignored. The list of the essential proteins of *E. coli* comes from database DEG, which contains 296 essential genes. 291 out of 296 essential genes are mapped to 254 distinct proteins which present in the PPI data of *E. coli*. In our study, these 254 proteins are considered as essential proteins of *E. coli* while other 2473(=2727–254) proteins are nonessential proteins of *E. coli*. The orthologous information of *E. coli* proteins is retrieved from InParanoid, by checking the counts that *E. coli* proteins have orthologs in the 99 reference organisms. Therefore, 1422 out of 2727 proteins have orthologs in at least one of reference organisms. 216 out of 254 essential proteins are included in these 1422 proteins.

**Figure 10:**
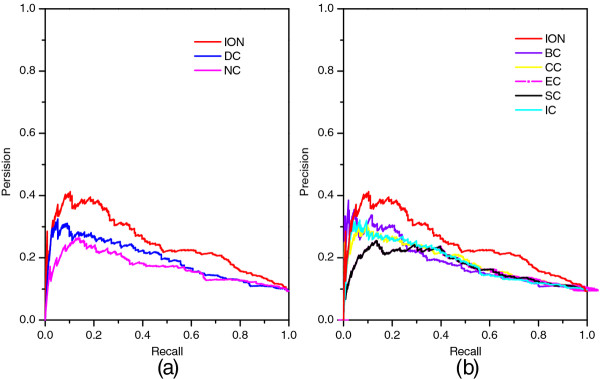
**PR curves of ION and seven other centrality methods based on protein data from*E. coli*.** The prediction performance of ION and seven other existing centrality methods (DC, BC, CC, SC, EC, IC and NC) based on protein data from *E. coli* are validated by the PR method.

**Figure 11:**
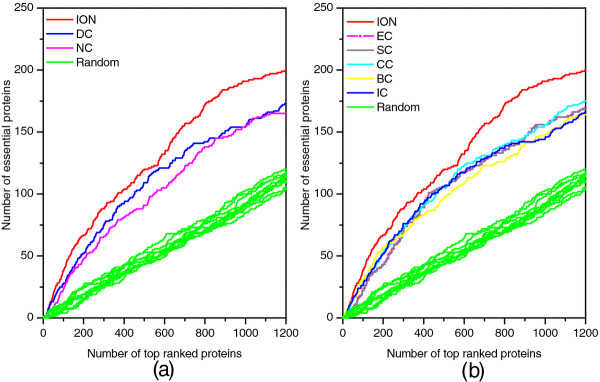
**Jackknife curves of ION and seven other centrality methods based on protein data from*E. coli*.** The prediction performance of ION and seven other existing centrality methods (DC, BC, CC, SC, EC, IC and NC) based on protein data from *E. coli* are validated by the jackknife method.

The ranking scores of *E. coli* proteins are calculated by using of ION (α=0.5) and the seven other existing centrality methods (DC, BC, CC, SC, EC, IC and NC), respectively. The number of essential proteins in top 1%, 5%, 10%, 15%, 20% and 25% of proteins ranked by these methods are listed in Table 6. The PR curves and jackknife curves of each method are illustrated in Figures 10 and 11. We do not compare ION with PeC because it requires gene express data of *E. coli*. All of these experimental result shows that the performance of ION in predicting essential proteins is better than that of the seven other existing centrality methods. Specially, as selecting top 10% and 25% ranked proteins, ION achieves 33% and 23% improvement than the average result of the seven methods, respectively.

**Table 6:** **Number of essential proteins identified by ION and seven other existing centrality methods based on protein data from*E. coli***

***K***	**DC**	**IC**	**EC**	**SC**	**BC**	**CC**	**NC**	**ION**
1%(27)	8	7	2	2	9	7	3	8
5%(136)	38	36	34	34	40	36	35	51
10%(273)	69	68	60	60	65	67	60	85
15%(409)	94	95	93	93	84	92	82	104
20%(545)	116	112	110	110	103	113	94	122
25%(682)	129	127	124	124	120	130	118	153

This table shows the comparison of the number of essential proteins identified by ION and seven other existing centrality methods (DC, BC, CC, SC, EC, IC and NC) based on protein data from *E. coli*. Since the total number of ranked proteins in *E. coli* is 2727. The number of proteins ranked in top 1% is about 27(=2727*1%). The digits in brackets denote the number of proteins ranked in each top percentage.

## Conclusions

Essential proteins play a key role in the life activities of cells. In this work we propose ION, an iteration method for predicting essential proteins based on orthology and PPI networks. In contrast to supervised machine learning methods, this method requires no prior knowledge of some reported essential proteins. Differently from centrality methods, ION identifies essential proteins depending on not only the connections between proteins but also their orthologous properties and features of their neighbors, which can overcome the limitation of the unreliability of PPI network data. Based on yeast PPI data, orthologs data and the data of known essential proteins, we firstly analyze the correlation between the essentiality of proteins and the counts that the proteins have orthologs in reference organisms. We further study the probability distribution of essential proteins in orthologs with respect to each available organism. From statistic data, we confirm the evolutionary conservation of essential proteins. In order to evaluate the performance of ION, we carry out experiments on yeast proteins data and assign proteins orthologous score based on 99 organisms. Experimental results show that (1) ION performs much better prediction of essential proteins than the eight other existing centrality methods. (2) ION is able to identify many essential proteins with low-connectivity ignored by the eight other existing centrality methods. (3) In top 100 of ranked proteins, ION can not only detect more essential proteins ignored by the eight other existing centrality methods but also exclude a large number of nonessential proteins which can’t be ignored by these methods. (4) More proteins in top 100 ranked by ION are essential proteins but also belong the complexes with certain biological functions. (5) In order to predict essential proteins accurately, we should select as many as possible reference organisms. (6) Considering the effect of α on ION, the smaller the value of α, the faster ION can converge, yet the lower the prediction accuracy of ION is. From experiments we suggest the optimum α value is 0.5. In the final part of this paper we show that in the prediction of essential proteins of *E. coli*, ION also outperforms the other seven existing centrality methods.

All kinds of experiment data indicate that integrating the orthology with PPI networks can indeed provide better performance in prediction of essential proteins. It confirms that there is a close relationship between the essentiality and both network connectivity and evolutionarily conserved properties of proteins. With more resources of orthologs being available, we can conveniently use the information of orthologs to predict essential proteins of other species by integrating PPI network data. The weighted PPI networks constructed by ION can be decomposed into the modules by using some methods [49,50]. As these modules include proteins both conserved and essential, this can give us a new insight for the research of biology evolution and conserved function modules. Additionally, ION can also provide us a framework to identify essential proteins by integrating biological properties with PPI network. By using ION, we can identify essential proteins and modules with certain biological functions by using other biological properties of essential proteins instead of their conserved properties.

## Competing interests

The authors declare that they have no competing interests.

## Authors’ contributions

WP obtained the protein-protein interaction data, essential proteins and Orthologous data. WP and JXW designed the new method, ION. WP and LQ analyzed the results. WP, JXW and FXW discussed extensively about this study and drafted the manuscript together. WPW and YP participated in revising the draft. All authors have read and approved the final manuscript.

## Supplementary Material

Additional file 1 **Algorithm convergence.** This file provides the proof of the algorithm convergence and the discussion about the effect of parameter α and ε on the speed of convergence. Click here for file

Additional file 2 **Proteins in top 100 ranked by ION while ignored by eight other existing centrality methods.** This file provides the list of proteins in top 100 ranked by ION while ignored by eight other existing centrality methods: Degree Centrality (DC), Betweenness Centrality (BC), Closeness Centrality (CC), Subgraph Centrality (SC), Eigenvector Centrality (EC), Information Centrality (IC), Edge Clustering Coefficient Centrality (NC) and centrality based on edge clustering coefficient and pearson correlation coefficient (PeC). In column Essential, the values “1” or “0” mean the proteins are either essential or nonessential. The values in column Ortholog_counts represent the counts that the proteins have orthologs in 99 referent organisms. The columns ranging from ION to PeC represent the ranking orders of the proteins in the results of corresponding methods. Click here for file

Additional file 3 **Top 100 proteins identified by ION and eight other centrality measures.** This file is composed by the lists of the top 100 proteins identified by ION and eight other centrality measures (DC, BC, CC, SC, EC, IC, NC and PeC). Furthermore, according to known complex lists, the proteins in top 100 ranked by ION, PeC, NC and DC are annotated with the index of the complexes that they belong to, but also these complexes are also annotated with their functions. Click here for file

Additional file 4 **Information about how to select reference organisms.** This file provides the detailed information how to select the 10, 20, 40, 60, 80, 90 reference organisms, when we discuss the effect of the number of reference organisms on the performance of ION in section “Discussions on the orthologous score”. In column ION_10, ION_20, ION_40, ION_60, ION_80, ION_90, the values “1” denote the organism is one of 10, 20, 40, 60, 80, 90 reference organisms, respectively. Click here for file

## Author details

^1^School of Information Science and Engineering, Central South University, Changsha, Hunan 410083, People’s Republic of China. ^2^Computer Technology Application Key Lab of Yunnan Province, Kunming University of Science and Technology, Kunming, Yunnan 650093, People’s Republic of China. ^3^Department of Mechanical Engineering and Division of Biomedical Engineering, University of Saskatchewan, Saskatoon SK S7N 5A9, Canada.^4^Department of Computer Science, Georgia State University, Atlanta, GA 30302-4110, USA.
