# Supplementary material for: Iteration method for predicting essential proteins based on orthology and protein-protein interaction networks
Source: BMC Syst Biol. 2012 Jul 18;6:87. doi: 10.1186/1752-0509-6-87 (PMC3472210; doi:10.1186/1752-0509-6-87)
Supplement: Additional file 1 — Algorithm convergence. This file provides the proof of the algorithm convergence and the discussion about the effect of parameter α and ε on the speed of convergence. [file 1752-0509-6-87-S1.pdf]

**Algorithm convergence**

THEOREM 1. Given matrix *H* and vector *d*, the algorithm converges. The number of iteration depends on parameters $\alpha$ and $\varepsilon$.

PROOF. The convergence of the algorithm depends on Equation (6). Equation (6) converges if and only if $\rho(\alpha H) < 1$, where $\rho(\alpha H)$ is the spectral radius of matrix $\alpha H$. Since $h(i, j) \geq 0$ and for each row i of matrix H, either $\sum_{j \in Ne(i)} h(i, j) = 1$ or $\sum_{j \in Ne(i)} h(i, j) = 0$, $\rho(H) = \|H\|_1 = 1$. It is easy to see that $\rho(\alpha H) = \alpha\rho(H) < 1$ for $0 \leq \alpha < 1$.

Therefore, Equation (6) converges and so does the algorithm. Furthermore, according to Equation (6),

$$pr^t = (1-\alpha)d + \alpha H * pr^{t-1}$$
$$pr^{t-1} = (1-\alpha)d + \alpha H * pr^{t-2}$$

And thus, we have

$$pr^t - pr^{t-1} = \alpha H(pr^{t-1} - pr^{t-2}) = \cdots = (\alpha H)^{t-1}(pr^1 - pr^0) = (\alpha H)^{t-1}\left[\alpha(H - I)d\right]$$

Therefore

$$\left\|pr^t - pr^{t-1}\right\|_1 = \left\|(\alpha H)^{t-1}(pr^1 - pr^0)\right\|_1 = \left\|(\alpha H)^{t-1}[\alpha(H - I)d]\right\|_1 \leq \alpha^t \left\|(H - I)d\right\|_1 \leq \varepsilon,$$

and

$$t \geq \log[\varepsilon / \|(H - I)d\|_1] / \log \alpha$$

Clearly, given matrix *H* and vector *d*, the iteration times *t* depends on parameter $\alpha$ and $\varepsilon$.

To investigate the influence of the parameter $\alpha$ and $\varepsilon$ on the number of iterations which are needed to converge, firstly, taking $\varepsilon = 10^{-8}$ we compare the number of iterations required to converge by setting different values of $\alpha$, ranging from 0.5 to 0.999. The results are tabulated in Table 1. As shown in Table 1, for $\alpha$ =0.5, the

algorithm needs only 28 iterations to converge to a tolerance of $10^{-8}$. For $\alpha$ =0.8, the number of iterations increases to 80. For $\alpha$ =0.999, the number of iterations rise sharply up to 1615, which is about 58 times larger than for $\alpha$ =0.5. So parameter $\alpha$ can indeed control the convergence rate of the algorithm.

To investigate the influence of the parameter $\varepsilon$ on the convergence, we compare the iterations by setting $\varepsilon$ different values ranging from $10^{-2}$ to $10^{-13}$ for $\alpha$ = 0.5, 0.7, 0.8, 0.9, respectively. Figure 1 shows that no matter what the value of $\alpha$ is, with the decrease of $\varepsilon$ the number of iterations increases slowly. When the value of $\varepsilon$ is $10^{-13}$ , which is $10^{-11}$ times smaller than $10^{-2}$ , the number of iterations required for convergence only increases about 4 times. Compared with $\alpha$ , the effect of $\varepsilon$ on the iteration is smaller. In fact, $\varepsilon$ controls the precision of *pr* values. The smaller the $\varepsilon$ value is, the more precise the *pr* values are. In order to distinguish the ranking scores of proteins, a suitable value of $\varepsilon$ should be set. However, the exact values of the *pr* vector are not as important as the correct ordering of the values in the vector. Moreover, the algorithm needs more time to converge, if the value of $\varepsilon$ is too small. Compromising the convergence of the algorithm and the precision of *pr* values, the parameter $\varepsilon$ is set as $10^{-8}$ in this study.

**Table 1 - Effect of parameter $\alpha$ on the number of iterations**

| $\alpha$ | 0.5 | 0.6 | 0.7 | 0.8 | 0.9 | 0.99 | 0.999 |
|---|---|---|---|---|---|---|---|
| number of iterations | 28 | 37 | 52 | 80 | 160 | 900 | 1615 |

The table shows the relationship between the values of parameter $\alpha$ and the number of iterations required to converge.

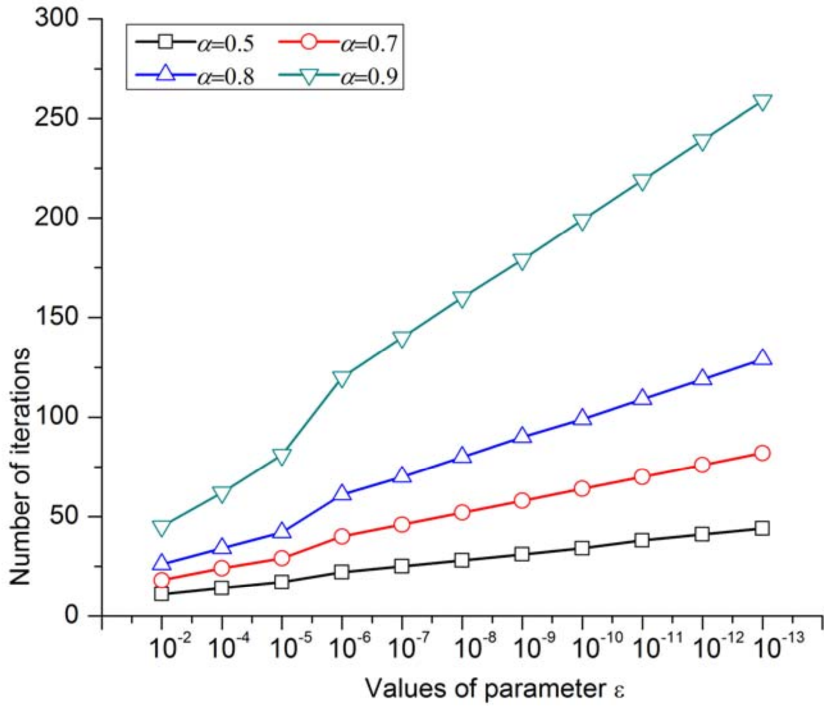


**Figure 1 - Number of iterations needed for ION convergence with different parameter $\varepsilon$ .**

X-axis represents the values of parameter $\varepsilon$ . Y-axis represents the number of iterations needed for ION convergence.
